# Apical periodontitis and its effects on renal tissue in rats

**DOI:** 10.17843/rpmesp.2024.414.13947

**Published:** 2024-11-25

**Authors:** Cynthia Mireya Jara, Roccio Raquel Ramírez, Regina Susana Barreto, Héctor García-Salinas, Carlos Gabriel Adorno, Vicente Fretes, Shyrley Paola Amarilla, Clarisse Díaz-Reissner

**Affiliations:** 1 National University of Asuncion, Faculty of Dentistry, Asuncion, Paraguay. National University of Asuncion National University of Asuncion Faculty of Dentistry Asunción Paraguay; 2 National University of Asuncion, Faculty of Medical Sciences, Asuncion, Paraguay. National University of Asuncion National University of Asuncion Faculty of Medical Sciences Asunción Paraguay; 3 National University of Asuncion, Faculty of Veterinary Sciences, San Lorenzo, Paraguay. National University of Asuncion National University of Asuncion Faculty of Veterinary Sciences San Lorenzo Paraguay

**Keywords:** chronic kidney disease, glomerulonephritis, endodontic inflammation, endodontics, blood pressure, Wistar rats, animal models

## Abstract

**Objectives.:**

To evaluate the effect of apical periodontitis (AP) induced in Wistar rats on histologically examined renal tissue.

**Materials and methods.:**

Fourteen 12-week-old male Wistar rats weighing an average of 250 grams were used. AP was induced with pulp exposure of the upper and lower first molars using a #1011 HL spherical bur in high rotation. The lesions were left exposed to the oral environment for a period of 7 weeks. Blood pressure was measured by the tail-cuff plethysmography method from the fourth week. The kidney was dissected for histological analysis (H&E). Mann-Whitney and Student’s t-test were used for non-parametric and parametric data, respectively, with a significance level of 5%.

**Results.:**

A statistically significant increase in both Bowman’s space area and renal corpuscle area was found in the AP group (p<0.05). The AP group had a higher percentage of renal tissue with inflammatory infiltrate, but without significant difference. Blood pressure did change during the experimental period and no difference was identified between the groups.

**Conclusions.:**

Induction of AP in Wistar rats resulted in significant changes of certain renal histological parameters, suggesting a possible interaction between AP and renal tissue that requires further research.

## INTRODUCTION

In recent decades, research in oral health has gained relevance due to increasing evidence demonstrating its intrinsic relationship with systemic health ^1^. There are several mechanisms by which oral pathologies such as chronic periodontitis can have an impact on distant organs. In addition to being associated with bacteremia and systemic inflammation, during the course of chronic periodontitis, bacteria present in periodontal tissues can spread through different pathways, including the hematogenous, oropharyngeal and oral-digestive routes ^2^. There is strong evidence correlating periodontal infections with an increased risk of cardiovascular disease ^3^.

Another highly prevalent oral pathology in the world population is apical periodontitis (AP). According to recent reports, half of the adults in the world have at least one tooth with AP ^4^. AP is the local inflammation of the periapical tissues that originates from an infection in the pulp chamber and is characterized by inflammation and subsequent destruction of the periradicular tissue ^5^. As with chronic periodontitis, endodontic disease or AP can lead to translocation of microorganisms from the root canal to the systemic environment, triggering immune responses that can affect other tissues or organs ^6^.

Previous research has reported that AP is associated with high levels of various interleukins and immunoglobulins ^7^^,^^8^. Contrary to previous beliefs, lesions of endodontic origin transcend the consideration of only having local repercussions. Although a definitive causal relationship has not yet been demonstrated ^9^, several studies suggest the existence of a moderate risk and correlation between certain systemic diseases and endodontic pathology ^10^^,^^11^. Renal pathologies can present as nephron lesions, which comprises the glomerulus, composed of a ball of glomerular capillaries and Bowman’s capsule that forms the initial urinary space, as well as the renal tubules, including the proximal, loop of Henle, distal and collecting tubules. In addition, these conditions can affect the interstitial tissue surrounding the nephron and the adjacent blood vessels ^12^.

Glomerular and tubulointerstitial conditions related to infectious and inflammatory processes have been identified. Membranoproliferative glomerulonephritis (MPGN) and tubulointerstitial nephritis stand out. MPGN is characterized by a glomerular pattern with mesangial hypercellularity and thickening of the basement membrane, associated to immunocomplex deposits and cytokine release. It is linked to several infections, including viral, chronic bacterial infections such as endocarditis, mycoses, and parasitic infections. Related microorganisms include *Staphylococcus*, *Mycobacterium*, *Streptococcus*, among others ^13^.

Tubulointerstitial nephritis is a renal lesion characterized by the presence of inflammatory infiltrates and tubulitis in the interstitial compartment ^14^, which can be acute or chronic. Several infectious processes, both bacterial and viral, can trigger the development of this condition ^15^. Although the exact pathogenic mechanism is not yet fully understood, it is speculated that it could involve the direct cytopathic effect of microorganisms or the release of proinflammatory cytokines during infection, which could act as inducers of renal damage ^12^.

These two conditions can cause chronic changes, with the subsequent development of chronic kidney disease (CKD) ^16^. During the evolution of CKD there is a gradual destruction of nephrons and a progressive loss of renal functions caused mainly by inflammation and activation of the immune system ^17^. Periodontitis, as well as intestinal dysbiosis, oxidative stress and acidosis, chronic and recurrent infections, increased production of proinflammatory cytokines and alterations in adipose tissue metabolism are currently considered factors influencing such dysregulation ^18^.

Given that both chronic periodontitis and AP share a chronic-inflammatory nature and are related to increased production of proinflammatory cytokines, it is reasonable to consider the possibility that chronic AP is also associated with renal disorders. In a recent case-control study involving 105 patients with CKD and 105 healthy patients, a significantly higher prevalence of AP was found in the group of patients with CKD. The authors suggest an association between AP severity and CKD markers, raising the possibility that AP may influence disease progression. However, they emphasize that since this was an observational study, their findings cannot establish a definitive causal relationship ^19^.

Considering that animal model studies allow more efficient identification of cause-effect relationships without the interference of confounding or uncontrolled variables ^20^, our study aimed to evaluate the effect of AP induced in Wistar rats on renal tissue. Additionally, we measured systolic BP in all animals during the last weeks of the experimental period.

**KEY MESSAGES**

Motivation for the study. Apical periodontitis (AP) can trigger immune responses that affect other organs.

Main findings. This animal study examined the effects of AP on renal tissue, finding significant changes in parameters such as renal corpuscle area and Bowman’s space, which may have implications for chronic kidney disease.

Implications. Future research will provide insight into how dental conditions may affect renal health. If confirmed, regular dental checkups would not only be critical to improve the overall health of patients with kidney disease, but could also serve as a preventive measure.

## MATERIALS AND METHODS

### Study design

Experimental study in laboratory animals designed to evaluate the effects of induced AP on renal tissue. Fourteen male Wistar rats, 12 weeks old and weighing approximately 250 grams, obtained from the biotherium of the Health Sciences Research Institute in Paraguay, were used. The animals were randomly divided into two groups: a control group (Group 1) and another with induced AP (Group 2). The animals were kept in the vivarium at the Physiopathology laboratory of the Faculty of Medical Sciences, National University of Asuncion, at a controlled temperature (24 ± 2 °C) and a 12-h light-dark cycle. Filtered water and standard commercial ration (porcine growth ration) were provided *ad libitum* that met the requirements of the species. The animals were kept and distributed in suitable and easy to clean cages. The bedding was covered with autoclaved wood shavings that were renewed twice a week.

For calculating the sample, we used data from a previous study of similar methodology that evaluated renal alterations in rats with induced periodontitis as reference ^23^. We used the mean and standard deviation of the renal corpuscle area in rats without periodontitis (4532.16 ± 313.77) and with periodontitis (7490.25 ± 2195.45). Using the GPower program (version 3.1), with a significance level of 5% and a power of 95%, the calculation indicated that 7 animals per group would be sufficient.

### Induction of apical periodontitis

All experimental procedures were performed under anesthesia with a combination of ketamine hydrochloride 90 mg/kg (Dopalem brand, Paulínia, SP, Brazil) and xylazine hydrochloride 10 mg/kg (Anasedam brand, Paulínia, SP, Brazil), administered intraperitoneally. After each procedure the animals underwent a recovery period in the laboratory, during which they were observed in order to detect any behavioral changes before returning to their respective cages. Paracetamol (300 mg/kg) was administered orally every 12 h during the first 24 h post-procedure.

In order to induce AP, the pulp in the mesial fossa of the upper and lower first molars was exposed using a #1011 HL spherical bur in high rotation (KGSorensen, Cotia, SP). A #10 gauge endodontic file (Dentsply Maillefer, Ballaigues, Switzerland) was used for removing debris from the pulp tissue, thus minimizing the possibility of causing painful symptoms in the postoperative period. The teeth were left exposed to the oral environment for a period of four weeks ^24^.

### Evaluation of systolic blood pressure

Blood pressure was measured by the tail-cuff plethysmography method (tail-cuff method) (CODA, Kent Scientific, Torrington, CT, USA). Operating procedures were carried out according to the manufacturer’s manual. Before measurement, each rat was placed on the thermal platform (35°C) of the equipment for 15 to 20 minutes to promote vasodilatation of the tail artery and facilitate the blood pressure measuring process. Measurements were taken 15 times, restricting to a maximum of 40 minutes for the entire procedure. To avoid blood pressure fluctuation as a result of circadian rhythms, data were collected between 9 to 12 m ^25^. A weekly blood pressure measurement was obtained from the fourth week onwards, taking into account that from this point onwards the periapical lesions in the rats are considered to have reached a chronic state ^24^.

### Specimen processing and histological analysis of the kidney

**Figure 1:**
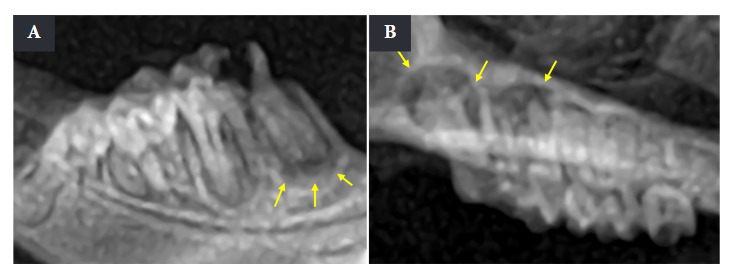
Radiographs of periapical lesions. A) Periapical lesion in the mandibular first molar. B) Periapical lesion in the maxillary first molar. The yellow arrows indicate the limits of each lesion.

Euthanasia was performed after seven weeks of AP induction using the intracardiac puncture technique, and the periapical status was reviewed with control radiographs (Figure 1). The radiological limits of the periapical lesions were observed in the AP group. Given the limited dimension of the oral cavity in the animal model, taking radiographs under live conditions is extremely complicated. For this reason, all radiographic evaluations for the verification of periapical lesion formation were performed post-euthanasia.

A midline incision was made in the thoracic region. The kidney was dissected and fixed in 10% formalin-buffered saline for 48 hours. Tissues were routinely processed and embedded in kerosene. Three 5 μm thick sectional sections were prepared per slide and then stained with hematoxylin and eosin (H & E). The stained sections were examined with a microscope and digital photomicrographs (Obj. 20X - 40X). All histological analyses were performed by an expert examiner and blinded regarding groups (SPA). The quantitatively analyzed parameters were: number of glomeruli (mm^2^), glomerulus area (µm^2^), glomerulus diameter (µm), renal corpuscle area (µm^2^), Bowman’s space area (µm^2^), Bowman’s space thickness (µm), basement membrane thickness (µm), glomerulus circumference (µm) and Bowman’s capsule circumference (µm). In addition, the presence/absence of inflammatory infiltrate was analyzed dichotomously.

### Statistical analysis

Shapiro-Wilk tests were performed to check the normality of the data for all the variables. The Mann-Whitney U test and Student’s t test were used for nonparametric and parametric data, respectively. Fisher’s exact test was used for the analysis of inflammatory infiltrate. The IBM SPSS version 25 statistical package was used for all analyses, with a significance level of 5%.

### Ethical considerations

This study was structured according to the guidelines established by the Guide to International Principles for Biomedical Research Involving Animals ^21^^)^ in addition to being in accordance with the essential guidelines established by the ARRIVE declaration ^22^. The protocol was approved by the Ethics Committee of the Faculty of Dentistry of the National University of Asuncion (Report 010/2021).

## RESULTS

No animal was lost during the experimental period. The average final weight of the control group was 387.42 ± 38.22 grams, while the animals of the AP group had an average final weight of 379.42 ± 25.97 grams.

A statistically significant increase was found in two of the nine analyzed renal parameters, Bowman’s space area and renal corpuscle area in the control group compared to AP (Table 1).

**Table 1:** Histological parameters of renal tissue of Wistar rats analyzed quantitatively.

Histological parameters	Control group	Group with apical periodontitis	p-value
	Mean ± SD	Mean ± SD	
Glomeruli	8.14 ± 3.67	8.71 ± 3.50	0.771^a^
Glomerulus diameter (µm)	75.16 ± 10.28	78.15 ± 4.51	0.495^a^
Bowman’s space area (µm^2^)	764.61 ± 192.12	1208.27 ± 243.95	0.003^a^
Bowman’s space thickness (µm)	3.87 ± 0.78	4.35 ± 1.16	0.384^a^
Basement membrane thickness (µm)	1.64 ± 0.37	1.91 ± 0.27	0.151^a^
Glomerulus circumference (µm)	236.00 ± 32.29	245.38 ± 14.17	0.495^a^
Circumference of Bowman’s capsule (µm)	248.15 ± 33.87	259.04 ± 16.43	0.459^a^
Glomerulus area (µm^2^)	4583.17 (3750.20 - 5383.76)^c^	5098.84 (4779.91 - 7045.94)^c^	0.073^b^
Renal corpuscle area (µm^2^)	5579.46 (4667.49 - 6364.83)^c^	6258.87 (5938.27 - 8459.87)^c^	0.026^b^

The effect size for the Bowman space area parameter was d = 2.02, indicating a large effect.

The effect size calculated for the renal corpuscle area parameter was r = -0.60, indicating an effect of moderate magnitude.

SD = standard deviation; ^a^ Student’s t test; ^b^ Mann-Whitney U test; ^c^ Median (minimum-maximum).

**Figure 2:**
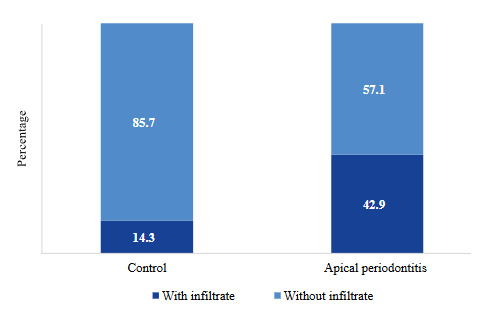
Presence of inflammatory infiltrate in renal tissue (n=14).

In the control group, 85.7% (n=6) of the samples showed no inflammatory infiltrate and 14.3% (n=1) showed inflammatory infiltrate. In the AP group, 57.1% (n=4) of the samples didn’t show infiltrate, and 42.9% (n=3) showed inflammatory infiltrate (Figure 2) (Fisher’s test; p=0.559).

**Figure 3:**
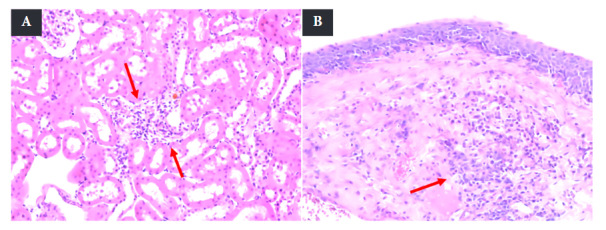
Inflammatory infiltrate of the kidney in mice with apical periodontitis. A. Superficial cortex. B. Pelvis. Arrows indicate the presence of polymorphonuclear cells (H&E, 20X).

A focus of mononuclear infiltrate in the cortex was found in some samples of the AP group, but other animals from the same group showed an inflammatory infiltrate of polymorphonuclear cells of varying intensity and diffuse distribution in the pelvis (Figure 3).

No significant difference was found between the groups regarding blood pressure measurements taken from the fourth to the seventh week (Mann-Whitney U; p>0.05) (Table 2).

**Table 2:** Evaluation of systolic blood pressure (mmHg) by group (n=14).

Week	Group	Median	IQR (Q1 - Q3)	(Min. - Max.)	p-value
4	Control	143.78	(129 - 156)	(113 - 158)	0.535
	AP	144.33	(139 - 152)	(127 - 166)	
5	Control	140.22	(131 - 147)	(128 - 146)	0.128
	AP	128.73	(126 - 137)	(119 - 156)	
6	Control	127.20	(108 - 137)	(88 - 158)	0.209
	AP	150.13	(123 - 154)	(117 - 152)	
7	Control	112.43	(92 - 123)	(78 - 132)	0.902
	AP	115.50	(103 - 127)	(90 - 128)	

AP: apical peridiontitis; IQR: interquartile range.

## DISCUSSION

As in our study, other authors have examined the possible relation between the presence of chronic periodontitis and its effects on renal structure and function. Recently, it has been suggested that chronic periodontitis should be considered as a frequent comorbidity in patients with CKD ^26^. On the other hand, it has also been suggested that periodontal treatment can have a positive impact on the glomerular filtration rate in individuals with CKD, which translates into renal function improvement ^27^. The correlation between CKD and periodontitis has also been supported by biomarker analyses in blood and saliva ^28^. Despite the available evidence, the mechanisms by which chronic periodontitis promotes progression to CKD is still not entirely clear ^29^^,^^30^.

There is evidence of progress in the field of periodontics research regarding its possible connections to systemic diseases. However, in the field of endodontics, although notable progress has been made, the number of studies linking AP with systemic level effects, particularly renal repercussions, are still limited. After a review of the related literature, it is possible to perceive that our study is one of the first to assess the relation between AP and its renal repercussions, through histological analysis in Wistar rats.

Significant increases in parameters associated with renal corpuscle area and Bowman’s space area were found in the group with induced AP. Although cellular proliferation and basement membrane thickening lesions have not been identified yet that allow classifying it as MPGN, it cannot be ruled out that this is the beginning of such condition. It is feasible to think that, in time, these characteristic lesions of MPGN may appear as the disease progresses. Lesions involving glomerular inflammation have been evidenced in certain autoimmune or infectious diseases ^12^. Thus, both chronic and acute renal diseases present a shared underlying mechanism involving inflammation and activation of the immune system, independently of the triggering cause ^17^.

Our findings show the presence of a higher percentage of renal tissue with inflammatory infiltration in the samples of animals in which AP was induced, although no statistically significant differences were found. This suggests the possibility of an onset of tubulointerstitial nephritis. Scientific literature supports the notion that autoimmune or infectious inflammatory processes may be underlying causes of this renal pathology ^12^^,^^31^. On the other hand, the common final outcome in most progressive chronic kidney diseases is renal fibrosis ^32^. There are several profibrotic factors involved in renal inflammation, including but not limited to TNF-α, IL-33, TGF-β, CCR1 and CCR2 ^33^.

According to studies performed in animal models and in humans, both symptomatic and asymptomatic AP can cause an increase in the levels of molecular markers of inflammation ^7^^,^^8^^,^^34^. The expression of CCR1 and CCR2 chemokine receptors has been detected in periapical cysts and granulomas, indicating their involvement in the pathogenesis of the disease ^35^. These inflammatory markers are also involved in the process of renal injury (membranoproliferative glomerulonephritis and tubulointerstitial nephritis), as well as in renal fibrosis secondary to these processes, suggesting a plausible biological basis for the relationship between AP and possible renal damage.

It is important to note that this study has certain methodological limitations that should be mentioned. In particular, the lack of additional complementary analyses, due to the unavailability of resources at the time, could have limited the depth of the results and the understanding of our findings. It would be important for future research to include complementary studies, such as the evaluation of glomerular injury by detecting alterations in glomerular barrier function and cell proliferation in the glomerulus or tubulointerstitial, assessed through the presence of proteinuria, hematuria, and leukocyturia, together with measurements of renal function by plasma creatinine concentration.

Furthermore, it would be beneficial for future research, to carry out similar studies for longer periods of time. Long-term follow-up of the animals could provide a more complete picture of the evolution of AP-induced renal changes. This would allow to determine whether the effects found in this study are reversible or progressive over time, which, together with the suggested complementary analyses, would contribute to a deeper understanding of the nature of the association between AP and kidney damage.

In conclusion, AP induction in Wistar rats resulted in a significant increase in parameters related to renal corpuscle area and Bowman’s space area. No relevant BP modifications were identified between groups during the experimental period.
