# Supplementary material for: Apical periodontitis and its effects on renal tissue in rats
Source: Rev Peru Med Exp Salud Publica. 2024 Nov 25;41(4):385–91. doi: 10.17843/rpmesp.2024.414.13947 (PMC11797580; doi:10.17843/rpmesp.2024.414.13947)
Supplement: Translation

# Translation: Periodontitis apical y sus efectos sobre el tejido renal en ratas

**Authors:** Cynthia Mireya Jara, Roccio Raquel Ramírez, Regina Susana Barreto, Héctor García-Salinas, Carlos Gabriel Adorno, Vicente Fretes, Shyrley Paola Amarilla, Clarisse Díaz-Reissner

**Affiliations:** 1 Universidad Nacional de Asunción, Facultad de Odontología, Asunción, Paraguay.; 2 Universidad Nacional de Asunción, Facultad de Ciencias Médicas, Asunción, Paraguay.; 3 Universidad Nacional de Asunción, Facultad de Ciencias Veterinarias, San Lorenzo, Paraguay.

**Keywords:** enfermedad renal crónica, glomerulonefritis, inflamación endodóntica, endodoncia, presión arterial, ratas Wistar, modelos animales

## Abstract

**Objetivos.:**

Evaluar el efecto de la periodontitis apical (PA) inducida en ratas Wistar sobre el tejido renal examinado histológicamente.

**Materiales y métodos.:**

Fueron utilizadas 14 ratas Wistar macho, de 12 semanas y 250 gramos promedio. La PA fue inducida con la exposición pulpar de los primeros molares superiores e inferiores utilizando una fresa esférica #1011 HL en alta rotación. Las lesiones se dejaron expuestas al medio bucal por un periodo de 7 semanas. La presión arterial fue medida mediante el método de pletismografía del manguito de la cola a partir de la cuarta semana. Fue realizada la disección del riñón para análisis histológico (H&E). Fue utilizada la prueba de Mann-Whitney y t de Student para datos no paramétricos y paramétricos, respectivamente, con un nivel de significancia del 5%.

**Resultados.:**

Se observó un aumento estadísticamente significativo tanto en el área del espacio de Bowman como en el área del corpúsculo renal en el grupo PA (p<0,05). El grupo con PA presentó mayor porcentaje de tejido renal con infiltrado inflamatorio, pero sin diferencia significativa. La presión arterial no sufrió modificaciones durante el periodo experimental y no fue identificada diferencia entre los grupos.

**Conclusiones.:**

La inducción de PA en ratas Wistar resultó en modificaciones significativas de ciertos parámetros histológicos renales, sugiriendo una posible interacción entre la PA y el tejido renal que requiere de investigaciones adicionales.

## INTRODUCCIÓN

En las últimas décadas, las investigaciones en la salud oral han adquirido relevancia debido al aumento de la evidencia que demuestra su intrínseca relación con la salud sistémica ^1^. Existen diversos mecanismos mediante los cuales las patologías bucales, como la periodontitis crónica pueden tener un impacto en órganos distantes. Además de estar asociada con bacteriemias e inflamación sistémica, durante el curso de la periodontitis crónica, las bacterias presentes en los tejidos periodontales pueden diseminarse a través de diferentes vías, incluyendo la vía hematógena, orofaríngea y orodigestiva ^2^. Existe evidencia sólida que correlaciona las infecciones periodontales con un mayor riesgo de enfermedades cardiovasculares ^3^.

Otra patología bucal altamente prevalente en la población mundial es la periodontitis apical (PA). De acuerdo con recientes reportes, la mitad de los adultos en el mundo tiene al menos un diente con PA ^4^. La PA es la inflamación local de los tejidos periapicales que se origina a partir de una infección en la cámara pulpar y se caracteriza por la inflamación y la consiguiente destrucción de los tejidos perirradiculares ^5^. De la misma manera que ocurre con la periodontitis crónica, la enfermedad endodóntica o PA puede provocar la translocación de microorganismos del conducto radicular al entorno sistémico, lo que desencadena respuestas inmunitarias que pueden afectar a otros tejidos u órganos ^6^.

Investigaciones previas han informado que la PA se encuentra asociada con niveles elevados de diversas interleucinas e inmunoglobulinas ^7^^,^^8^. Contrariamente a las creencias previas, las lesiones de origen endodóntico trascienden la consideración de ser únicamente de repercusión local. Aunque aún no se ha demostrado una relación causal definitiva ^9^, diversos estudios sugieren la existencia de un riesgo moderado y una correlación entre ciertas enfermedades sistémicas y la patología endodóntica ^10^^,^^11^. Las patologías renales pueden presentarse como lesiones de la nefrona, la cual comprende el glomérulo, compuesto por un ovillo de capilares glomerulares y la cápsula de Bowman que forma el espacio urinario inicial, así como los túbulos renales, incluyendo el proximal, el asa de Henle, el distal y el colector. Además, estas patologías pueden afectar el tejido intersticial circundante a la nefrona y los vasos sanguíneos adyacentes ^12^.

Se han identificado patologías glomerulares y tubulointersticiales relacionadas con procesos infecciosos e inflamatorios. Se destacan la glomerulonefritis membranoproliferativa (GNMP) y las nefritis tubulointersticiales. La GNMP se caracteriza por un patrón glomerular con hipercelularidad mesangial y engrosamiento de la membrana basal, asociado a depósitos de inmunocomplejos y liberación de citoquinas. Está vinculada a diversas infecciones, incluyendo virales, bacterianas crónicas como endocarditis, micosis y parasitarias. Los microorganismos relacionados incluyen *Staphylococcus, Mycobacterium, Streptococcus*, entre otros ^13^.

La nefritis tubulointersticial es una lesión renal que se caracteriza por la presencia de infiltrados inflamatorios y tubulitis en el compartimento intersticial ^14^, pudiendo presentarse en forma aguda o crónica. Diversos procesos infecciosos, tanto bacterianos como víricos, pueden desencadenar el desarrollo de esta afección ^15^. Aunque el mecanismo patogénico exacto aún no se comprende completamente, se especula que podría involucrar el efecto citopático directo de los microorganismos o la liberación de citocinas proinflamatorias durante la infección, que podrían actuar como inductores del daño renal ^12^.

Estas dos patologías pueden provocar cambios crónicos, con el posterior desarrollo de enfermedad renal crónica (ERC) ^16^. Durante la evolución de la ERC existe una destrucción gradual de las nefronas y una pérdida progresiva de las funciones renales causados principalmente por la inflamación y activación del sistema inmunológico ^17^. La periodontitis, así como la disbiosis intestinal, estrés oxidativo y acidosis, infecciones crónicas y recurrentes, una mayor producción de citocinas proinflamatorias y alteraciones del metabolismo del tejido adiposo son actualmente considerados factores que influyen en dicha desregulación ^18^.

Dado que tanto la periodontitis crónica como la PA comparten una naturaleza crónico-inflamatoria y están relacionadas con un aumento en la producción de citocinas proinflamatorias, es razonable considerar la posibilidad de que la PA crónica también esté asociada con trastornos renales. En un estudio de caso-control reciente que involucró a 105 pacientes con ERC y 105 pacientes sanos, se observó una prevalencia significativamente mayor de PA en el grupo de pacientes con ERC. Los autores sugieren una asociación entre la gravedad de la PA y los marcadores de la ERC, lo que plantea la posibilidad de que la PA pueda influir en la progresión de la enfermedad. Sin embargo, destacan que al tratarse de un estudio observacional, estos hallazgos no establecen una relación causal definitiva ^19^.

Teniendo en cuenta que los estudios en modelo animal permiten identificar más eficientemente relaciones causa-efecto sin la interferencia de variables de confusión o no controladas ^20^, el presente estudio tiene por objetivo evaluar el efecto de la PA inducida en ratas Wistar sobre el tejido renal. Adicionalmente, se llevó a cabo la evaluación de la presión arterial sistólica de todos los animales durante las últimas semanas del período experimental.

**MENSAJE CLAVE**

Motivación para el estudio. La periodontitis apical (PA) puede desencadenar respuestas inmunitarias que afectan otros órganos.

Principales hallazgos. Este estudio en animales examinó los efectos de la PA en el tejido renal, encontrando cambios significativos en parámetros como el área del corpúsculo renal y el espacio de Bowman, indicando posibles implicaciones para la enfermedad renal crónica.

Implicancias. Futuras investigaciones permitirán comprender cómo las afecciones dentales pueden afectar la salud renal. De confirmarse, los controles dentales periódicos no solo serían fundamentales para mejorar la salud integral de los pacientes con enfermedad renal, sino que también podrían servir como una medida preventiva.

## MATERIALES Y MÉTODOS

### Diseño del estudio

Estudio experimental en animales de laboratorio diseñado para evaluar los efectos de la PA inducida sobre el tejido renal. Fueron utilizadas 14 ratas Wistar macho, de 12 semanas de vida y con un peso aproximado de 250 gramos obtenidos del bioterio del Instituto de Investigaciones en Ciencias de la Salud en Paraguay. Los animales se dividieron aleatoriamente en dos grupos: uno de control (Grupo 1) y otro con PA inducida (Grupo 2). Los animales se mantuvieron en el vivario dependiente del laboratorio de Fisiopatología de la Facultad de Ciencias Médicas-Universidad Nacional de Asunción, a temperatura controlada (24 ± 2 °C) y ciclo de luz oscuridad de 12 h. Se proporcionó agua filtrada y ración comercial estándar (ración porcina crecimiento) *ad libitum* que cumplía con los requisitos de la especie. Los animales se mantuvieron y distribuyeron en jaulas adecuadas y fáciles de limpiar. El lecho fue cubierto de virutas de madera previamente esterilizadas por autoclave que fueron renovadas dos veces por semana.

El cálculo de la muestra se realizó utilizando como referencia los datos de un estudio previo de metodología similar que evaluó las alteraciones renales en ratas con periodontitis inducida ^23^. Los datos extraídos y utilizados para el cálculo fueron la media y desviación estándar del área del corpúsculo renal en ratas sin periodontitis (4532,16 ± 313,77) y con periodontitis (7490,25 ± 2195,45). Utilizando el programa GPower (versión 3.1), con un nivel de significancia del 5% y una potencia del 95%, el cálculo indicó que 7 animales por grupo serían suficientes.

### Inducción de la periodontitis apical

Todos los procedimientos experimentales se realizaron bajo anestesia con una combinación de clorhidrato de ketamina de 90 mg/kg (marca Dopalem, Paulínia, SP, Brasil) y clorhidrato de xilacina 10 mg/kg (marca Anasedam, Paulínia, SP, Brasil), administrada por vía intraperitoneal. Al final de cada procedimiento, los animales se sometieron a un período de recuperación en el laboratorio, donde fueron observados por cualquier cambio de comportamiento antes de regresar a sus respectivas jaulas. Durante las primeras 24 h posteriores al procedimiento, se administró paracetamol (300 mg/kg) por vía oral cada 12 horas.

Para la inducción de la PA fue realizada la exposición pulpar en la fosa mesial de los primeros molares superiores e inferiores utilizando una fresa esférica #1011 HL en alta rotación (KGSorensen, Cotia, SP). Se empleó una lima endodóntica de calibre #10 (Dentsply Maillefer, Ballaigues, Suiza) con el propósito de remover los residuos del tejido pulpar, minimizando de esta manera la posibilidad de provocar síntomas dolorosos en el período postoperatorio. Los dientes se dejaron expuestos al ambiente bucal durante un período de cuatro semanas ^24^.

### Evaluación de la presión arterial sistólica

La presión arterial fue medida mediante el método de pletismografía del manguito de la cola (método tail-cuff) (CODA, Kent Scientific, Torrington, CT, EE. UU.). Los procedimientos operativos se llevaron a cabo de acuerdo con el manual del fabricante. Antes del inicio de la medición, cada rata fue colocada sobre la plataforma térmica (35° C) del equipo durante 15 a 20 minutos para promover la vasodilatación de la arteria de la cola y facilitar el proceso de lectura de la presión arterial. Las mediciones se tomaron 15 veces, restringiendo a máximo 40 minutos para todo el procedimiento. Para evitar la fluctuación de la presión arterial como resultado de los ritmos circadianos, los datos se recopilaron entre las 9 a 12 m ^25^. Se realizó una medición semanal de la presión arterial a partir de la cuarta semana, teniendo en cuenta que a partir de este punto se considera que las lesiones periapicales en las ratas han alcanzado un estado crónico ^24^.

### Procesamiento de muestras y análisis histológico del riñón

La eutanasia se realizó luego de siete semanas de la inducción de la PA mediante la técnica de punción intracardiaca, y se revisó el estado periapical con radiografías de control (Figura 1). En el grupo PA se observaron los límites radiológicos de las lesiones periapicales. Dada la limitada dimensión de la cavidad oral en el modelo animal utilizado, la realización de radiografías en condiciones vivas se presenta como una tarea extremadamente complicada. Por esta razón, todas las evaluaciones radiográficas para la verificación de la formación de las lesiones periapicales se llevaron a cabo post-eutanasia.

**Figura 1:** Radiografías de las lesiones periapicales. A) Lesión periapical en el primer molar mandibular. B) Lesión periapical en el primer molar maxilar. Las flechas amarillas indican los límites de cada lesión.AB

Se realizó una incisión en la línea media en la región torácica. El riñón se diseccionó y se fijó en solución salina con formalina al 10% durante 48 horas. Los tejidos se procesaron de forma rutinaria y se incluyeron en parafina. Se prepararon tres cortes seccionales de 5 μm de espesor por portaobjetos y luego se colorearon con hematoxilina y eosina (H & E). Las secciones teñidas se examinaron con un microscopio y fotomicrografías digitales (Obj. 20X - 40X). Todos los análisis histológicos fueron realizados por un examinador experto y ciego con respecto a los grupos (SPA). Los parámetros analizados de manera cuantitativa fueron: número de glomérulos (mm^2^), área del glomérulo (µm^2^), diámetro del glomérulo (µm), área del corpúsculo renal (µm^2^), área del espacio de Bowman (µm^2^), grosor del espacio de Bowman (µm), grosor de la membrana basal (µm), circunferencia del glomérulo (µm) y circunferencia de la cápsula de Bowman (µm). Además, fue analizada de manera dicotómica la presencia/ausencia de infiltrado inflamatorio.

### Análisis estadístico

Para comprobar la normalidad de los datos de todas las variables mencionadas, se realizaron pruebas de Shapiro-Wilk. Fue utilizada la prueba de U de Mann-Whitney y t de Student para datos no paramétricos y paramétricos, respectivamente. Para el análisis del infiltrado inflamatorio fue utilizada la prueba exacta de Fisher. Para todos los análisis se utilizó el paquete estadístico IBM SPSS versión 25, con un nivel de significancia del 5%.

### Consideraciones éticas

El trabajo fue estructurado siguiendo las directrices establecidas por la Guía de Principios Internacionales para Investigaciones Biomédicas en animales ^21^ además de encontrarse en conformidad con los lineamientos esenciales establecidos por la declaración ARRIVE ^22^. El protocolo contó con la aprobación del Comité de Ética de la Facultad de Odontología de la Universidad Nacional de Asunción (Informe 010/2021).

## RESULTADOS

Durante el periodo experimental, no se registró pérdida de ningún animal. El peso final promedio del grupo control fue de 387,42 ± 38,22 gramos, mientras que los animales del grupo PA presentaron un peso final promedio de 379,42 ± 25,97 gramos.

En dos de los nueve parámetros renales analizados se observó un aumento estadísticamente significativo, estos fueron el área del espacio de Bowman como en el área del corpúsculo renal en el grupo control comparado con PA (Tabla 1).

**Tabla 1:** Parámetros histológicos del tejido renal de ratas Wistar analizados cuantitativamente

Parámetros histológicos	Grupo control	Grupo con periodontitis apical	Valor de p
	Media ± DE	Media ± DE	
Glomérulos	8,14 ± 3,67	8,71 ± 3,50	0,771^a^
Diámetro del glomérulo (µm)	75,16 ± 10,28	78,15 ± 4,51	0,495^a^
Área del espacio de Bowman (µm^2^)	764,61 ± 192,12	1208,27 ± 243,95	0,003^a^
Grosor del espacio de Bowman (µm)	3,87 ± 0,78	4,35 ± 1,16	0,384^a^
Grosor de la membrana basal (µm)	1,64 ± 0,37	1,91 ± 0,27	0,151^a^
Circunferencia del glomérulo (µm)	236,00 ± 32,29	245,38 ± 14,17	0,495^a^
Circunferencia de la cápsula de Bowman (µm)	248,15 ± 33,87	259,04 ± 16,43	0,459^a^
Área del glomérulo (µm^2^)	4583,17 (3750,20 - 5383,76)^c^	5098,84 (4779,91 - 7045,94)^c^	0,073^b^
Área del corpúsculo renal (µm^2^)	5579,46 (4667,49 - 6364,83)^c^	6258,87 (5938,27 - 8459,87)^c^	0,026^b^

El tamaño del efecto para el parámetro área del espacio Bowman fue d = 2,02, indicando un efecto grande.

El tamaño del efecto calculado para el parámetro área de corpúsculo renal fue r = −0,60, indicando un efecto de magnitud moderada.

DE = desviación estándar; ^a^ T de Student; ^b^ U de Mann-Whitney; ^c^ Mediana (mínimo- máximo)

En el grupo control, el 85,7% (n=6) de las muestras no presenta infiltrado inflamatorio, mientras que el 14,3% (n=1) sí lo presenta. En el grupo PA, el 57,1% (n=4) de las muestras están sin infiltrado, y el 42,9% (n=3) muestran infiltrado inflamatorio (Figura 2) (Prueba de Fisher; p=0,559).

**Figura 2:** Presencia de infiltrado inflamatorio en el tejido renal (n=14).

En algunas muestras del grupo PA, se pudo observar la presencia de un foco de infiltrado mononuclear en la corteza, mientras que, en otros animales del mismo grupo, se identificó la pelvis con un infiltrado inflamatorio de polimorfonucleares de intensidad variable y distribución difusa (Figura 3).

**Figura 3:** Infiltrado inflamatorio de riñón en ratón con periodontitis apical. A) Corteza superficial. B) Pelvis. Fechas señalando la presencia de células polimorfonucleares (H&E, 20X).

En cuanto a las mediciones de presión arterial realizadas desde la cuarta hasta la séptima semana, no fue encontrada diferencia significativa entre los grupos (U de Mann-Whitney; p>0,05) (Tabla 2).

**Tabla 2:** Evaluación de la presión arterial sistólica (mm Hg) por grupo (n=14).

Semana	Grupo	Mediana	RIC (Q1 - Q3)	(Mín. - Máx.)	Valor de p
4	Control	143,78	(129 - 156)	(113 - 158)	0,535
	PA	144,33	(139 - 152)	(127 - 166)	
5	Control	140,22	(131 - 147)	(128 - 146)	0,128
	PA	128,73	(126 - 137)	(119 - 156)	
6	Control	127,20	(108 - 137)	(88 - 158)	0,209
	PA	150,13	(123 - 154)	(117 - 152)	
7	Control	112,43	(92 - 123)	(78 - 132)	0,902
	PA	115,50	(103 - 127)	(90 - 128)	

PA: peridiontitis apical; RIC: rango intercuartílico

## DISCUSIÓN

Al igual que nuestro estudio, otros autores han examinado la posible relación entre la presencia de periodontitis crónica y sus efectos sobre la estructura y función renal. Recientemente, se ha indicado que la periodontitis crónica debería considerarse como una comorbilidad frecuente en pacientes con ERC ^26^. Por otro lado se ha sugerido que el tratamiento periodontal puede tener un impacto positivo en la tasa de filtrado glomerular en individuos con ERC, lo que se traduce en mejoras en su función renal ^27^. La correlación entre la ERC y la periodontitis también ha sido respaldada por análisis de biomarcadores en sangre y saliva ^28^. A pesar de la evidencia disponible, los mecanismos por los cuales la periodontitis crónica promueve la progresión a la ERC aún no es del todo clara ^29^^,^^30^.

En el campo de la periodoncia se evidencia un avance de la investigación con sus posibles conexiones a enfermedades sistémicas. No obstante, en el ámbito de la endodoncia, aunque se han logrado progresos notables, la cantidad de estudios que vinculan la PA con efectos a nivel sistémico, especialmente las repercusiones renales, son aún limitadas. Después de una revisión de la literatura relacionada, es posible percibir que el presente estudio es uno de los primeros en investigar la relación entre la PA y sus repercusiones renales, a través de análisis histológicos en ratas Wistar.

Se observaron incrementos significativos en los parámetros asociados con el área del corpúsculo renal y el área del espacio de Bowman en el grupo con PA inducida. Aunque aún no se han identificado lesiones de proliferación celular y engrosamiento de la membrana basal que permitan clasificarla como una GNMP, no se puede descartar que este sea el inicio de dicha patología. Es factible pensar que con el transcurso del tiempo estas lesiones características de la GNMP se manifiesten a medida que la enfermedad evolucione. En ciertas enfermedades autoinmunes o infecciosas, se han evidenciado lesiones que involucran inflamación glomerular ^12^. De esta manera, las enfermedades renales, tanto crónicas como agudas, presentan un mecanismo subyacente compartido que involucra la inflamación y la activación del sistema inmunológico, independientemente de la causa desencadenante ^17^.

Nuestros hallazgos evidencian la presencia de un mayor porcentaje de tejido renal con infiltración inflamatoria en las muestras de animales en los que se indujo la PA, aunque no se observaron diferencias estadísticamente significativas. Esto sugiere la posibilidad de un inicio de nefritis tubulointersticial. La literatura científica respalda la noción de que los procesos inflamatorios autoinmunes o infecciosos pueden ser causas subyacentes de esta patología renal ^12^^,^^31^. Por otro lado, el desenlace final común en la mayoría de las enfermedades renales crónicas progresivas es la fibrosis renal ^32^. Existen diversos factores profibróticos involucrados en la inflamación renal, incluyendo pero no limitándose a TNF-α, IL-33, TGF-β, CCR1 y CCR2 ^33^.

De acuerdo con estudios realizados en modelos animales y en seres humanos, la PA tanto sintomática como asintomática puede ocasionar un incremento de los niveles de marcadores moleculares de inflamación ^7^^,^^8^^,^^34^. Se ha detectado la expresión de receptores de quimiocinas CCR1 y CCR2 en quistes y granulomas periapicales, indicando su participación en la patogénesis de la enfermedad ^35^. Estos marcadores inflamatorios también están involucrados en el proceso de lesión renal (glomerulonefritis membranoproliferativa y nefritis tubulointersticial), así como en la fibrosis renal secundaria a estos procesos, lo que sugiere una base biológica plausible para la relación entre la PA y posibles daños renales.

Es importante señalar que este estudio presenta ciertas limitaciones metodológicas que deben mencionarse. En particular, la falta de análisis complementarios adicionales, debido a la no disponibilidad de recursos en ese momento, pudo haber limitado la profundidad de los resultados y la comprensión de los hallazgos obtenidos. Para futuras investigaciones, sería pertinente incluir estudios complementarios, como la evaluación de la lesión glomerular mediante la detección de alteraciones en la función de la barrera glomerular y la proliferación celular en el glomérulo o el tubulointersticial, evaluados a través de la presencia de proteinuria, hematuria y leucocituria, junto con mediciones de la función renal mediante la concentración de creatinina plasmática.

Además, sería beneficioso, en investigaciones futuras, considerar una extensión temporal del estudio. El seguimiento a largo plazo de los animales podría ofrecer una visión más completa de la evolución de los cambios renales inducidos por la PA. Esto permitiría determinar si los efectos observados en este estudio son reversibles o progresivos con el tiempo, lo que, junto con los análisis complementarios sugeridos, contribuiría a una comprensión más profunda de la naturaleza de la asociación entre la PA y el daño renal.

En conclusión, la inducción de PA en ratas Wistar resultó en un aumento significativo en los parámetros relacionados con el área del corpúsculo renal y el área del espacio de Bowman. No se identificaron modificaciones relevantes en la presión arterial entre los grupos durante el período experimental.
